# Strategies for optimal calorie administration in critically ill patients

**DOI:** 10.1186/s40560-019-0371-7

**Published:** 2019-03-12

**Authors:** Tomoaki Yatabe

**Affiliations:** Department of Anesthesiology and Intensive Care Medicine, Kochi Medical School, Kohasu, Oko-cho, Nankoku, Kochi 783-8505 Japan

**Keywords:** Calorie, Indirect calorimetry, Overfeeding, Underfeeding

## Abstract

Nutritional therapy is one of the important treatments in critically ill patients. How to estimate calorie consumption and how to determine an optimal calorie dose are clinical questions of great importance. Although indirect calorimetry is the gold standard for assessing energy expenditure, many intensivists are unable to use this technique. Therefore, the use of formulas, such as the Harris-Benedict equation, or the simple predictive value of 25 kcal/kg/day is reasonable. Several studies and guidelines have shown that the strategies for nutritional therapy depend on the nutritional risk of patients. If patients have low nutritional risks, these estimated values should not be adopted in the acute phase. Until the patient’s condition improves, less than 18 kcal/kg/day might be an optimal calorie target. Contrastingly, cumulative negative energy balance can also be harmful to critically ill patients. Thus, it is important to accurately determine the energy requirement and to make the required changes in the administered calorie dose to go from a strategy of “defense” to that of “offense” in a timely manner. In this article, the concepts of optimal calorie administration in critically ill patients were reviewed.

## Background

Nutritional therapy is one of the important treatments in critically ill patients. Several clinical practice guidelines have been established to help make decisions related to nutritional therapy [1–3]. The route, dose, and timing of nutrition are based on the patient’s condition. How to estimate calorie consumption and how to determine the optimal calorie dose are clinical questions of capital importance. In this article, we have discussed concepts of optimal calorie administration.

## Why is optimal calorie administration important?

Why is it necessary to think about optimal calorie administration before discussing the appropriate calorie dosage? For example, for treating an infectious disease, if vancomycin is not given at an appropriate dosage, there could be a risk of treatment failure due to under administration and a risk of kidney injury due to over administration. As with any antibiotic treatment, both over and under administration of calories are harmful to critically ill patients. In fact, a retrospective study conducted by Zusman et al. revealed that increasing the calorie administration/resting energy expenditure (REE) to 70% was associated with decreased mortality, while an increase above 70% was associated with increased mortality, especially an increase to > 100% [4]. Therefore, they concluded that both overfeeding and underfeeding might be harmful for critically ill patients [4].

Overfeeding is defined as energy administration of 110% above the defined target [3]. It is associated with hyperglycemia, hyperlipidemia, hypercapnia, infectious complications, impaired immunity, liver steatosis, and increased fat mass [5, 6]. Recently, impairment of autophagy caused by overfeeding is receiving a lot of attention [6, 7]. Because of the complex interplay among autophagy, immune responses, and inflammation [7], overfeeding should be avoided in critically ill patients. Contrastingly, underfeeding is defined as energy administration below 70% of the defined target [3]. It is associated with hypoglycemia, hypothermia, infectious complications, impaired immunity, impaired healing, loss of lean and fat body mass, and impaired muscle function [5]. Thus, ensuring optimal calorie administration is important in nutritional therapy.

## What do the guidelines say?

As per the guidelines of the Society of Critical Care Medicine (SCCM) and the American Society for Parenteral and Enteral Nutrition (ASPEN), nutritional risk should be determined for all patients admitted to the intensive care unit (ICU) [1]. This guideline defines low nutritional risk as Nutritional Risk Screening (NRS) 2002 ≤ 3 or Nutrition Risk in Critically ill (NUTRIC) scores ≤ 5 [1]. Because the NRS-2002 does not specialize in ICU patients, almost all ICU patients are considered at “risk” just because admission to the ICU (acute physiology and chronic health evaluation [APACHE] II score > 10) adds 3 points. Contrastingly, the NUTRIC score, based on age, APACHE II score, sequential organ failure assessment (SOFA) score, comorbidities, days from hospital admission to ICU admission, and interleukin (IL)-6 levels, identifies patients with scores ≥ 5 as having high risk [7, 8]. Since the measurement of IL-6 levels is not always feasible, a modified NUTRIC (mNUTRIC) score that excludes IL-6 levels is also used [9].

The SCCM/ASPEN guidelines described that specialized nutrition therapy during the first week of hospitalization in the ICU is not required for low-nutritional-risk patients [1]. Also, trophic or full nutrition via enteral nutrition (EN) is appropriate for patients with acute respiratory distress syndrome (ARDS) and for those expected to be on mechanical ventilation for ≥ 72 h [1]. In addition, administration of more than 80% of the estimated or calculated energy goal within 48–72 h is recommended for patients who are at high nutritional risk or are severely malnourished [1]. These guidelines also recommend that hypocaloric (≤ 20 kcal/kg/day or 80% of the estimated energy requirement) parenteral nutrition (PN) be considered in these patients if EN cannot be provided [1]. The Japanese guidelines recommend administration of the approximate number of calories involved in typical caloric intake during the initial ICU period for patients who are not malnourished [10]. Recent guidelines of the European Society for Clinical Nutrition and Metabolism (ESPEN) also recommend hypocaloric nutrition (below 70% of the estimated needs or energy expenditure [EE]) in the first week of ICU stay [3]. Thus, hypocaloric nutrition during the acute phase, namely the first week of ICU stay, is preferable in low-nutritional-risk patients.

## How to estimate energy needs?

**Fig. 1:**
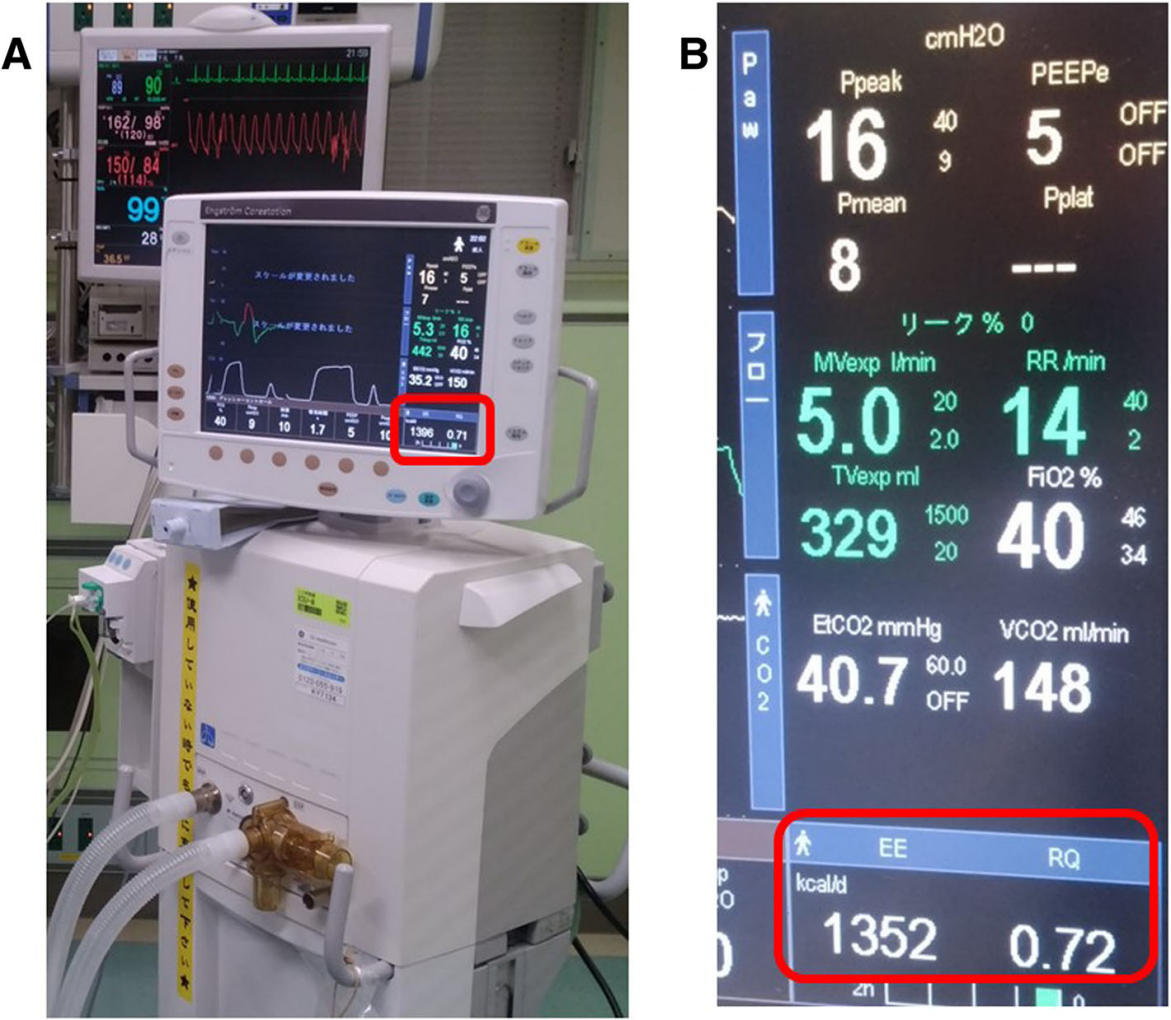
Indirect calorimetry. This device consists of a ventilator and an indirect calorimeter

As mentioned above, accurate determination of estimated energy needs or EE is essential for deciding the required energy dose. As per the SCCM/ASPEN guidelines, indirect calorimetry (IC) is recommended for determining energy requirements (Fig. 1). The use of a published predictive value or a simplistic weight-based value (25–30 kcal/kg/day) is also recommended in the absence of IC [1]. However, the ESPEN guidelines suggest that if IC is unavailable, EE should be determined using VO_2_ (oxygen consumption) from the pulmonary arterial catheter or VCO_2_ (carbon dioxide production) from the ventilator, which allows for better evaluation than the predictive values [3]. The EE is calculated using the following equation [11, 12]:

REE = CO × Hb (SaO_2_ − SvO_2_) × 95.18

Two previous studies have compared between EE determined using IC vs. VO_2_. Flancbaum et al. enrolled 36 ventilated surgical ICU patients [11] and reported that REEs measured by IC were significantly higher than those calculated using VO_2_ (2005 ± 464 vs. 1496 ± 590 kcal/kg/day). However, this method underestimated the REE in 83% of cases [11]. Another prospective study involving 40 ventilated surgical ICU patients [12] revealed that although the mean REE measured by IC was comparable to that calculated using VO_2_ (1928 ± 558 vs. 1989 ± 518 kcal/day), the REE differed by ≥ 20% in 70% of patients [12]. Since a pulmonary artery catheter is mandatory for measuring VO_2_, estimation of REE using VO_2_ might not be suitable for daily clinical situations.

**Fig. 2:**
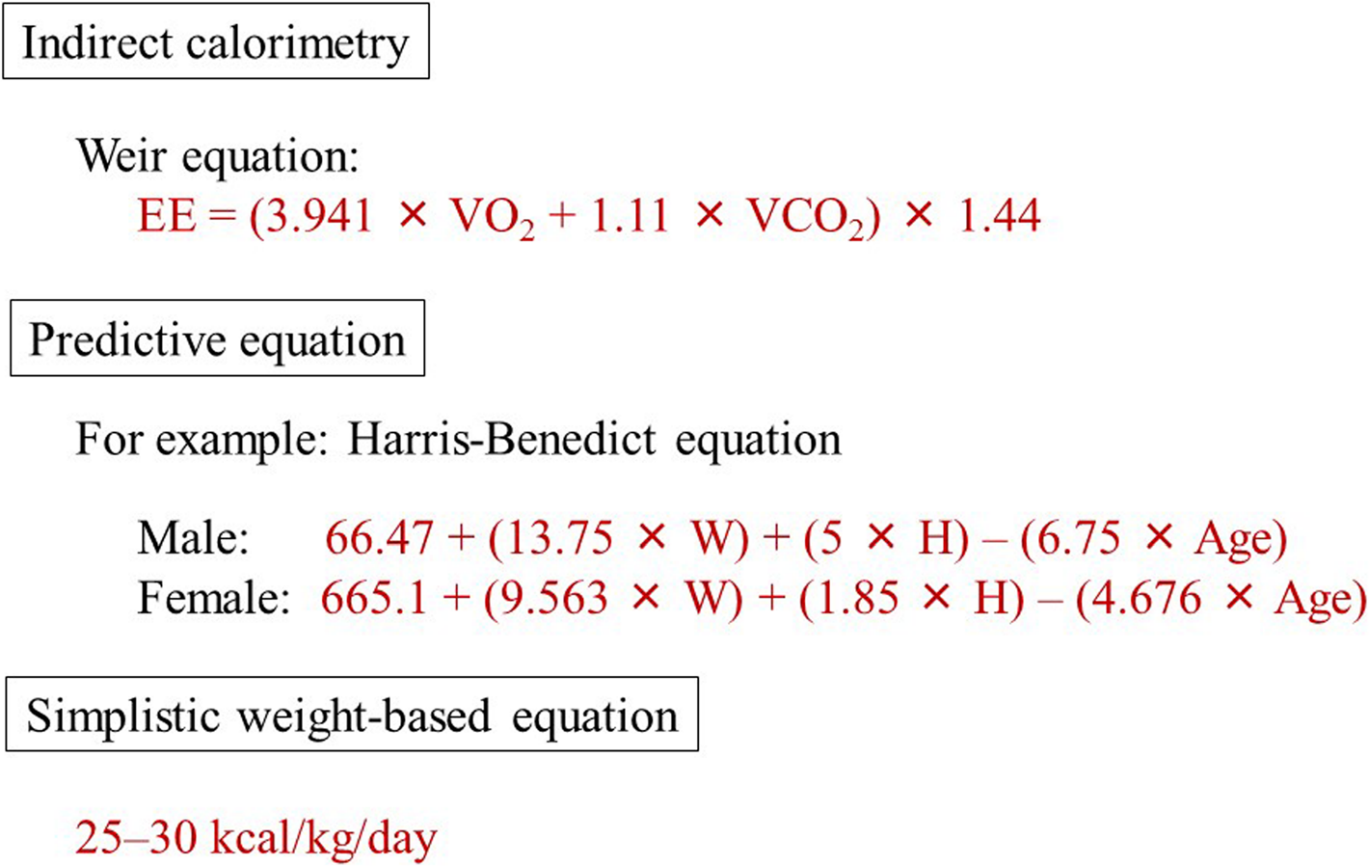
Methods of calculating or estimating energy expenditures. Indirect calorimetry measures VO_2_ and VCO_2_ from respiratory gases via masks or tracheal tubes. Then, energy expenditure (EE) is calculated using the Weir equation. In addition, the respiratory quotient (RQ) is calculated by dividing VCO_2_ by VO_2_. EE and RQ values are shown in Fig. 1. VO_2_ oxygen consumption, VCO_2_ carbon dioxide production, W weight, H height

In IC, VO_2_ and VCO_2_ are measured from respiratory gases. The EE is then calculated using the Weir equation [5] (Fig. 2):

$$ \mathrm{EE}=\left(3.941\times {\mathrm{VO}}_2+1.11\times {\mathrm{VCO}}_2\right)\times 1.44 $$

Several ventilators are able to measure VCO_2_. When VCO_2_ is known, the Weir equation can be used to calculate VO_2_, assuming the respiratory quotient (RQ), which is the ratio between VCO_2_ and VO_2_ [13]. Thus, the EE can be calculated without the VO_2_ value using the revised Weir equation:

$$ \mathrm{EE}=\left(3.941\times \left({\mathrm{VCO}}_2/\mathrm{RQ}\right)+1.11\times {\mathrm{VCO}}_2\right)\times 1.44 $$

Determination of the RQ value is a major problem in this method. A recent retrospective study enrolled 80 critically ill patients on ventilation to assess the accuracy of REE obtained from VCO_2_ [14]. In this study, the RQ values were arbitrarily chosen as 0.8, 0.85, and 0.89 because these values were the most commonly used for deriving REE from VO_2_ [14]. The results of the study showed that while 40–43% of the estimated values were within 85–115% of the measured REE, 13–18% of the estimated values were within 95–105% of the measured REE [14]. Contrastingly, another prospective study used nutritional RQ, which was calculated based on 24-h macronutrient delivery, in 84 ventilated ICU patients [13]. This study reported that although the EE calculated using VO_2_ with nutritional RQ was significantly higher than the REE measured using IC (1963 ± 431 vs. 1823 ± 408 kcal/kg/day, *p* < 0.001), less than 10% and 15% of the accuracy rates in VCO_2_-based EE were 61% and 79%, respectively [13]. Therefore, they concluded that EE assessment based on ventilator-derived VCO_2_ was accurate [13].

However, not all administered nutrients are absorbed in critically ill patients [15]. It is arguable whether VO_2_ is the most relevant variable for EE measurement [16]. Furthermore, a special ventilator that can measure VO_2_ is mandatory for this method. Thus, though IC is the gold standard for estimating energy needs in critically ill patients, the use of a predictive equation, such as the Harris-Benedict equation (HBE), or a simplistic weight-based value (25–30 kcal/kg/day) is feasible in situations where IC is not available.

## Is indirect calorimetry superior to other methods?

IC is the only practical clinical method for EE measurement and is considered as the gold standard [5, 17]. As described above, this technique measures VO_2_ and VCO_2_ for calculating EE using the Weir equation. Contrastingly, the HBE, one of the popular predictive equations, estimates EE using four simple factors, namely, gender, age, height, and weight [18]:

66.47 (13.75 × weight) + (5 × height) − (6.75 × age) for men and 665.1 + (9.563 × weight) + (1.85 × height) − (4.676 × age) for women

Several studies have been performed to evaluate EE estimation using the HBE in ICU settings (Table 1). Tatucu-Babet et al. conducted a systematic literature review to determine the prevalence of under prescription and over prescription of energy needs by comparing between the REE measured using predictive equations and using IC in critically ill patients [19]. In the review, they reported that the equations underestimated and overestimated REE in 38% and 12% of the cases, respectively, by more than 10% compared to IC measurements [19]. Subsequently, several studies have compared the use of IC and HBE in various ICU populations. Picolo et al. conducted an observational study including 205 ventilated critically ill patients [18], of which 44% had sepsis and 27% had pneumonia [18]. Although the REE measured by IC was comparable to that measured using the HBE, the HBE overestimated in the group with the REE measured by IC less than 1200 kcal/day and underestimated in the group with the REE measured by IC more than 1800 kcal/day [18]. Moreover, the Bland-Altman analysis revealed that REE calculated using the HBE was an overestimation by + 555.3 kcal/day and an underestimation by − 593.0 kcal/day [18]. Therefore, it was concluded that the HBE is not a reliable substitute for IC [18].

**Table 1:** Comparison between an indirect calorimetry and the Harris-Benedict equation

Author, year	Design No. of Hosp.	No. of Pt.	Type of patients	Age (years)	BMI (kg/m^2^)	Weight (kg)	APACHE II	SOFA	IC (kcal/day) (kcal/kg/day)	HBE (kcal/day)
Picolo et al. 2016 [18]	Prospective 1	205	Surgical 15% Sepsis 44%	54	18.5–24.9 42% 25.0–29.9 28%	71	24	NA	1430 NA	1463
Sabatino et al. 2017 [20]	Prospective 2	42	KDIGO stage 3 Surgical 52%	67 ± 15	29 ± 9	83 ± 26	22 ± 7	NA	1724 ± 431 NA	1582 ± 335
de Goes et al. 2016 [21]	Prospective 1	125	AKIN-3 Sepsis 89%	63 ± 17	28 ± 8	NA	29 ± 5	NA	2029 ± 760 NA	1501 ± 327
Panitchote et al. 2017 [22]	Prospective 1	16	Severe sepsis Septic shock	72 ± 6	22 ± 3	NA	27 ± 4	NA	1488 ± 261 26.7 ± 5.3	2259 ± 305*
Tignanelli et al. 2017 [23]	Prospective** 1	419	Surgical 100%	> 60 42%	18.5–24.9 25% 25.0–29.9 30%	NA	NA	NA	1837 ± 547 NA	1894 ± 354
Yatabe et al. 2014 [24]	Retrospective 1	15	Esophagectomy 100%	66 ± 10	22 ± 4	55 ± 10	13 ± 4	2 ± 1	985 ± 167 18.1 ± 3.4	1191 ± 159
De Waele et al. 2018 [25]	Prospective 1	7	ECMO Surgical 29%	64 [60, 77]	26 [25, 27]	78 [68, 90]	19 [13, 23]	NA	1334 21	NA
Segadilha et al. 2017 [26]	Retrospective 1	97	Elderly Surgical 28%	78 ± 9	27 ± 7	NA	NA	NA	1568 ± 374 M 22.6 ± 4.7 F 21.4 ± 5.1	(kcal/kg/day)*** M 22.4 ± 2.2 F 22.1 ± 3.0
Hickmann et al. 2014 [27]	Prospective 1	49	Sepsis 63%	53–71	NA	NA	15–22	5–11	1849 ± 546 NA	1433 ± 230

*Hosp.* hospital, *Pt.* patients, *BMI* body mass index, *IC* indirect calorimetry, *HBE* Harris-Benedict equation, *Prospective* prospective observational study, *NA* not available, *KDIGO* Kidney Disease Improving Global Outcomes classifications, *AKIN* Acute Kidney Injury Network classifications, *Retrospective* retrospective study, *M* male, *F* female

*Harris-Benedict equation × 1.6

**Retrospective review of prospectively collected data

***Harris-Benedict equation × 1.2

A prospective multicenter observational study was conducted to evaluate the validity of the HBE and the guideline-recommended value (25 kcal/kg/day) for calculating EE by using IC in 42 acute kidney injury (AKI) patients [20]. Of these 42 patients, 19 received renal replacement therapy (RRT) [20]. This study revealed that only 38% and 28% of the estimates calculated using the HBE and the recommended value of 25 kcal/kg/day, respectively, yielded optimal values, namely, values corresponding to 90–110% of the IC measurements [20]. In patients who received RRT, 47% of the estimates using the HBE were underestimated, while 79% of the estimates using the recommended value of 25 kcal/kg/day were overestimates [20]. Another prospective study compared the HBE and IC in 125 mechanically ventilated AKI patients [21]. This study reported that the REE calculated using IC was significantly higher than that estimated using the HBE (2029 ± 760 vs. 1501 ± 327 kcal/kg/day, *p* < 0.001), and only 18% of the cases had a predicted value that was within 10% of the REE measured using the HBE [21].

Panitchote et al. compared between the HBE and IC in 16 patients with severe sepsis and septic shock [22]. This single-center prospective observational study revealed that average REE calculated using IC over 72 h was 26.7 ± 5.3 kcal/kg/day; the Bland-Altman analysis revealed that the REE calculated using the HBE, when multiplied by 1.6 as a correction factor, was an overestimation by + 757 kcal/day [22].

Tignanelli et al. performed a retrospective review of prospectively collected data to compare between REE measured using IC and the HBE in 419 ventilated adult surgical ICU patients [23]. This study included critically ill adults who were mechanically ventilated for > 24 h for non-cardiothoracic and non-burn ailments [23]. Although the REE measured by IC was comparable to that estimated by the HBE (1837 ± 547 vs. 1894 ± 354 kcal/kg/day, *p* = 0.07), the percent accuracy within + 10% of the measured REE was 35% [23]. In addition, REE was overestimated using the guideline-recommended values of 25 kcal/kg/day and 30 kcal/kg/day (2178 ± 668 and 2614 ± 803 kcal/kg/day, respectively), with percent accuracies within + 10% of 25% and 11%, respectively [23]. Another previous retrospective study has compared REE measured by IC and that estimated by the HBE in postoperative ventilated patients who underwent minimally invasive esophagectomy [24]. Although this study only evaluated 15 patients and used IC until postoperative day 1, the average REE measured by IC was significantly lower than that estimated by the HBE (985 ± 167 vs. 1191 ± 159 kcal/day, 83 ± 10% of the HBE measurement, *p* < 0.001) [24].

A recent prospective study compared calculated EEs during extracorporeal membrane oxygenation (ECMO) treatment in seven stable patients [25]. This study revealed a median EE of 21 kcal/kg/day, although the range (12–33 kcal/kg/day) was wide [25]. Thus, they concluded that the HBE as well as the guideline-recommended value (25 kcal/kg/day) provided inappropriate metabolic information in patients receiving ECMO [25].

Segadilha et al. conducted a retrospective study to compare between REE measured by IC and by HBE (multiplied by 1.2 as a correction factor) in 97 critically ill elderly patients [26]. In this study, the average age was 77.9 ± 8.5 years, and 49% of the population was aged 80 years or older [26]. They reported that the REE measured by IC was comparable to that obtained by multiplying the HBE with a correction factor of 1.2 (22.6 ± 4.7 vs. 22.4 ± 2.2 kcal/kg/day) [26], whereas the use of the HBE without a correction factor might underestimate the REE. This study also reported that the guideline-recommended value of 25 kcal/kg/day overestimated the REE in 55.1% of men and 60.4% of women [26].

The influence of physical therapy on EE was evaluated by an observational study including 49 hemodynamically stable critically ill patients [27]. This study revealed that 30 min of cycling at 3 and 6 W increased the EE by 39.3 ± 16.3% and 54.1 ± 16.7%, respectively, while passive exercise did not increase the EE [27]. In addition, they reported that the REE determined by IC was higher than the REE determined by the HBE (29 ± 31%, *p* < 0.001) [27].

Based on the results of all these studies, the HBE and the guideline-recommended value cannot provide an accurate estimation of the REE. Recently, a randomized controlled trial (RCT) was performed to investigate whether nutrition therapy involving IC, instead of equations for assessment of energy needs, could improve the nutrition status in critically ill patients [28]. Forty patients who were on mechanical ventilation for at least 3 days and were expected to stay in the medical ICU for more than 2 days were enrolled in this RCT [28]. These patients were randomized into two groups: the IC group and the standard care (SC) group. Energy needs were repeatedly determined using IC in the IC group and were calculated once using the recommended value of 25 kcal/kg/day in the SC group [28]. Although the energy requirement in the IC group was significantly lower than that in the SC group (21.1 ± 6.4 vs. 25 kcal/kg/day, *p* < 0.01), mean intake was comparable in both groups (20.4 ± 5.7 vs. 20.0 ± 7.5 kcal/kg/day) [28]. This RCT also reported that the length of mechanical ventilation (9 ± 8 vs. 10 ± 5 days) and hospital mortality (25% vs. 15%) were also comparable in both groups [28]. Though IC is the gold standard for estimating REE, there is little evidence to support its positive effect on outcomes in critically ill patients. In addition, the answer to the basic clinical question “Is measured EE always reflective of the energy needs?” remains controversial [5]. The initial cost of the device used for IC might be a limiting factor in its widespread use [5]. For many intensivists who cannot use IC, the results of studies on IC, such as the ones discussed here, help in daily clinical practice. Although these aforementioned studies show that several factors, such as pathophysiological conditions, severity, and treatment, affect EE, the use of the guideline-recommended value of 25 kcal/kg/day might be reasonable. In fact, a recent multicenter observational study including 13 Japanese ICUs revealed that the median calorie target was 25.2 kcal/kg/day, comparable to the guideline-recommended value [29].

## What is the optimal calorie dose that can be administered?

After determining the optimal calorie target, namely the estimated or measured EE, the next clinical question is “How soon should it be administrated?”. Tian et al. performed a systematic review to compare between initial hypocaloric EN and hypercaloric EN in critically ill patients [30]. Of the eight RCTs included in the study, mean daily percentage of target calories was < 33.3% in two studies, 33.3–66.6% in four studies, and > 66.6% in two studies in the low-energy group, whereas it was > 70% in seven studies and 59.2% in one study in the high-energy group [30]. This systematic review reported no significant differences between the low-energy and high-energy groups in terms of mortality (relative risk [RR], 0.90; 95% confidence interval [CI], 0.71–1.15; *p* = 0.40), infections (RR, 1.09; 95% CI, 0.92–1.29; *p* = 0.32), or risk of gastrointestinal intolerance (RR, 0.84; 95% CI, 0.59–1.19; *p* = 0.33) [30]. Subgroup analysis revealed that mortality in the low-energy subgroup (33.3–66.6% of the energy target) was significantly lower than that in the high-energy group (RR, 0.68; 95% CI, 0.51–0.92; *p* = 0.01) [30]. After publication of this systematic review, Arabi et al. reported a large multicenter RCT to compare permissive underfeeding (40–60% of the calculated caloric requirements) with standard enteral feeding (70–100%) for up to 14 days [31]. This trial enrolled 894 patients; calorie intake in the permissive underfeeding group was significantly lower than that in the standard feeding group (835 ± 297 vs. 1299 ± 467 kcal/day, *p* < 0.001; 46 ± 14% vs. 71 ± 22% of caloric requirements, *p* < 0.001) [31]. Although 90-day mortality was comparable between the two groups (27.2% vs. 28.9%, *p* = 0.58), the incidence of RRT was significantly lower in the permissive underfeeding group than in the standard feeding group (7.1% vs. 11.4%, *p* = 0.04) [31]. It is noteworthy that the high-energy groups in five of the studies included in this systematic review and the standard feeding group in the RCT reported by Arabi et al. did not receive more than 80% of the energy target.

Recently, the results of a large RCT that assessed the effects of a large number of calories on 90-day mortality in patients on mechanical ventilation were published [32]. Patients in the ICU were administered 1.5 kcal/ml or 1.0 kcal/ml by EN for 28 days. The total calorie intake in the 1.5 kcal/ml group was higher than that in the 1.0 kcal/ml group (23.9 ± 7.8 vs. 17.4 ± 5.5 kcal/kg/day) [32]. However, the 90-day mortality was comparable in both the groups (26.8% vs. 25.7%), suggesting that energy intake did not affect the survival of critically ill adults [32].

Another recent systematic review evaluated eight RCTs involving 5360 critically ill patients to compare between the outcomes of PN + EN and EN alone [33]. Although hospital mortality was found to be comparable in both the groups (RR, 0.91; 95% CI, 0.74–1.12; *p* = 0.36), the risk of respiratory infections was significantly higher in the PN + EN group than in the EN alone group (RR, 1.13; 95% CI, 1.01–1.25; *p* = 0.03) [33]. Because the lack of information on caloric intake is an important limitation of this review, it does not provide clear evidence that permissive underfeeding might be beneficial. A meta-analysis by the ESPEN compared hypocaloric nutrition with isocaloric nutrition regardless of the route of administration [3] and found comparable incidence of mortality and infections in both the groups (RR, 1.01; 95% CI, 0.86–1.18; *p* = 0.93 and RR, 0.94; 95% CI, 0.84–1.05; *p* = 0.29, respectively) [3]. Based on this evidence, it is reasonable to conclude that hypocaloric nutrition (< 70% of the estimated need or EE) in the first week of ICU stay, as per the ESPEN guidelines, might be beneficial.

However, a recent review described that nutritional risk plays an important role in the selection of feeding strategies [7]. In fact, a multicenter prospective observational study was performed to investigate whether clinical outcomes vary according to energy intake in patients having nutritional risk, as determined by the NUTRIC score [34]. They reported that mortality decreased significantly by 11.6% in high-nutritional-risk patients (odds ratio [OR], 0.884; 95% CI, 0.829–0.941; *p* < 0.001) for every 10% increase in the target energy intake. However, this effect was not observed in low-nutritional-risk patients (OR, 1.067; 95% CI, 0.967–1.178; *p* = 0.194) [34]. A recent retrospective study aimed to identify the association between calorie adequacy and 30-day mortality in patients undergoing emergency abdominal surgery [35]. This study revealed that in the high mNUTRIC score group, patients with calorie adequacy of < 70% had higher 30-day mortalities than those with adequate calorie intakes (31.5% vs. 11.1%; *p* = 0.01); however, this relationship was not observed in patients with low mNUTRIC scores (6.3% vs. 18.2%; *p* = 0.07) [35]. According to these results, hypocaloric nutrition should not be adopted in high-nutritional-risk patients.

## Until when should underfeeding be continued in low-nutritional-risk patients?

**Fig. 3:**
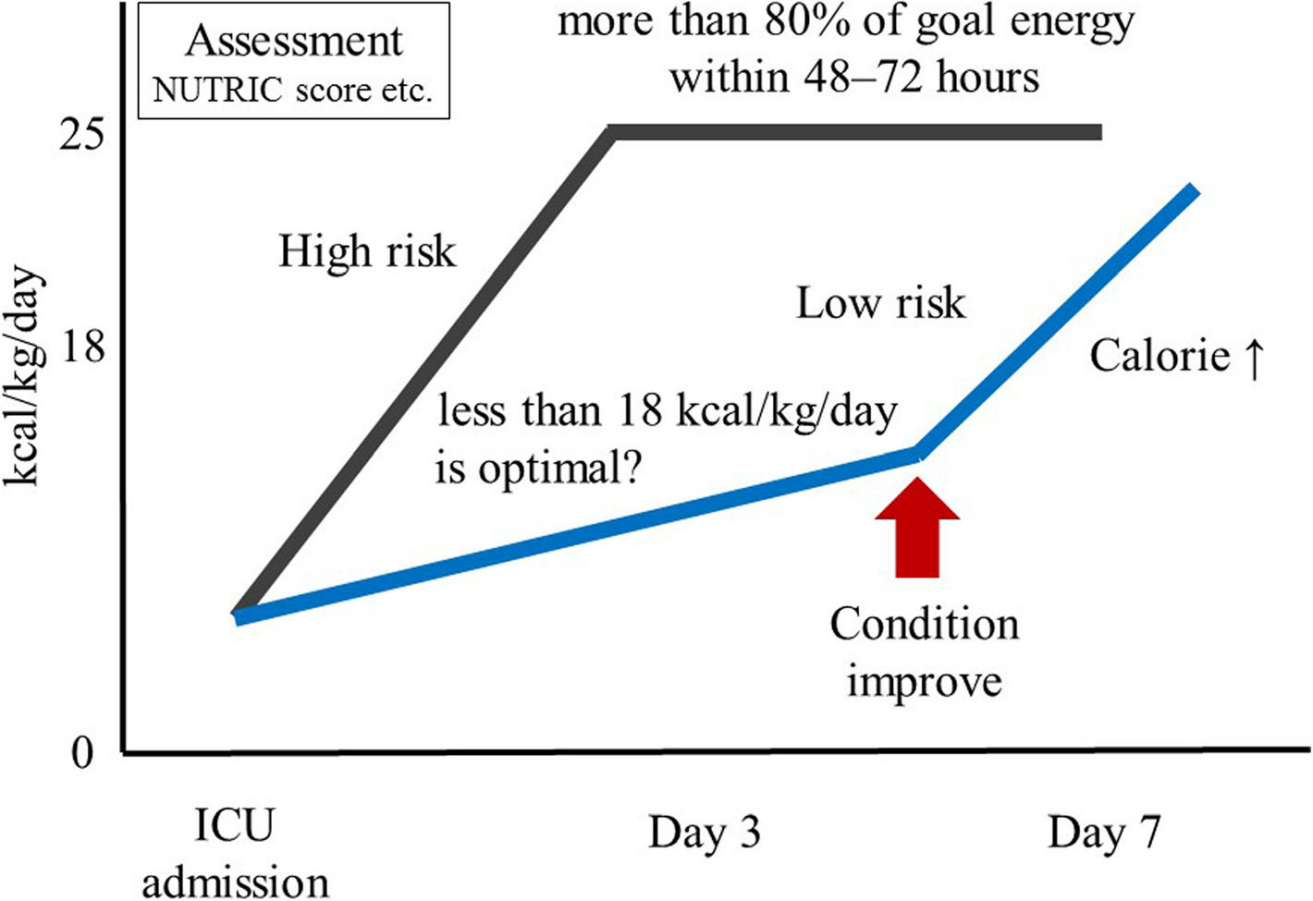
Strategies for optimal calorie administration in critically ill patients. When patients are admitted to the ICU, intensivists evaluate nutritional risk. If patients have high nutritional risk, more than 80% of the energy target is administered within 48–72 h of ICU admission. If patients have low nutritional risk, less than 18 kcal/kg/day might be an optimal calorie target (“defense” strategy). Then, after the patient’s condition starts improving, the administered calorie dose might be increased (“offense” strategy)

A previous prospective observational study demonstrated an association between cumulative negative energy balance and increasing number of complications, particularly infections [36], indicating that a prolonged strategy of underfeeding might be harmful. Thus, it is important to switch from the strategy of “defense” (underfeeding) to that of “offense” (adequate feeding) at the right time. In fact, post hoc analysis of an RCT including patients with acute lung injuries revealed that high-calorie intake until day 7 was associated with mortality (HR, 1.17; 95% CI, 1.07–1.28 for every 1 kcal increase/kg; *p* = 0.0004) whereas high-calorie intake after day 8 was associated with reduced mortality (HR, 0.91; 95% CI, 0.83–1.0; *p* = 0.04) [37]. Based on these results, a cutoff value of 18 kcal/kg/day was recommended [37]. Another prospective observational study in Japan found that a caloric intake of less than 10 kcal/kg/day on day 3 was associated with poor physical status at ICU discharge (OR, 1.19; 95% CI, 1.05–1.34; *p* = 0.005) whereas the same caloric intake on day 7 was not [29]. A recent single-center retrospective study explored the relationships of organ failure, SOFA score, and calorie intake with hospital mortality during the first week of ARDS [38]. Their results showed that an increase in SOFA score and average calorie intakes < 12 kcal/kg and ≥ 12 kcal/kg were associated with an incremental increase in mortality (OR, 2.27; 95% CI, 1.08–4.74; *p* = 0.03 and OR, 4.22; 95% CI, 2.02–8.78; *p* < 0.001, respectively) [38]. According to these studies and guidelines, it appears that estimated or calculated EE should not be adopted as the optimal dose of calorie administration in the acute phase if patients have low nutritional risk. In such cases, less than 18 kcal/kg/day might be an optimal calorie target. Additionally, although it is difficult to determine the right time for changing the nutritional strategy from “defense” to “offense”, it might be beneficial to increase the administrated calorie dose when the patient’s condition begins to improve (Fig. 3). Then, this timing might be decided based on the time when the SOFA scores start decreasing.

## Conclusion

In critically ill patients, the strategy for nutritional therapy depends on nutritional risk. Thus, intensivists should evaluate this risk when patients are admitted to the ICU. Practically, EE is estimated either by an equation, such as the HBE, or by using a simple predictive value of 25 kcal/kg/day. In low-nutritional-risk patients, this estimated value should not be adopted in the acute phase. It is also crucial that the change in administered calorie dose is made at the right time.
